# Acute myocardial infarction in a 16-year-old patient – A journey from death to life: Case report

**DOI:** 10.1097/MD.0000000000043975

**Published:** 2025-08-22

**Authors:** Yuke Xie, Ning Yang, Ping Zhang

**Affiliations:** a Department of Cardiovascular Medicine, Puyang Oilfield General Hospital Affiliated to Xinxiang Medical University, Puyang, Henan Province, China; b Department of Intensive Care Unit, Puyang Oilfield General Hospital Affiliated to Xinxiang Medical University, Puyang, Henan Province, China.

**Keywords:** 16 years old, acute myocardial infarction, death

## Abstract

**Rationale:**

Acute myocardial infarction (AMI) in young individuals has become increasingly prevalent in recent years, with the age of onset progressively declining. According to the China Acute Myocardial Infarction Registry, which included over 24,000 cases, approximately 8.5% of AMI patients were aged ≤45 years. However, AMI occurring in adolescents remains exceptionally rare. Early recognition and timely intervention in such patients pose significant clinical challenges and carry important implications for improving outcomes in this population.

**Patient concerns:**

We report the case of a 16-year-old previously healthy male who presented with persistent precordial pain lasting over 4 hours. On admission, an emergency electrocardiogram showed atrial fibrillation with a heart rate of 117 bpm, abnormal Q waves in leads I, aVL, V2, and V3, and ST segment elevations ranging from 0.1 to 0.8 mV in leads I, aVL, and V1–V5.

**Diagnoses:**

Based on clinical presentation and ECG findings, the patient was diagnosed with acute extensive anterior and high lateral wall myocardial infarction.

**Interventions:**

Emergency coronary angiography revealed total occlusion of the left main coronary artery. The patient was immediately treated with venoarterial extracorporeal membrane oxygenation (VA-ECMO) and received comprehensive supportive care.

**Outcomes:**

Despite aggressive intervention, the patient developed catastrophic intracranial hemorrhage and subsequently died.

**Lessons:**

This case highlights the importance of considering AMI in the differential diagnosis of chest pain even in adolescents, and underscores the need for heightened clinical awareness, early diagnostic evaluation, and rapid therapeutic decision-making. Further research is needed to understand the underlying mechanisms and risk factors contributing to early-onset AMI in young individuals.

## 1. Introduction

Acute myocardial infarction (AMI) is a cardiovascular condition characterized by the blockage of coronary arteries, leading to sustained ischemia and hypoxia and ultimately resulting in myocardial necrosis. Among cardiovascular diseases, AMI is considered one of the most sudden and life-threatening events. In recent years, the age of onset of AMI has shown a clear trend toward younger patients. According to the China Acute Myocardial Infarction Registry, which included 24,000 patients, 8.5% of those diagnosed with AMI were aged 45 years or younger.^[1,2]^ Further analysis identified smoking, hypertension, obesity, diabetes, and hyperlipidemia as the most common risk factors.^[3]^ This report describes the case of a 16-year-old male patient who suffered an AMI and ultimately died.

## 2. Case introduction

**Figure 1.**
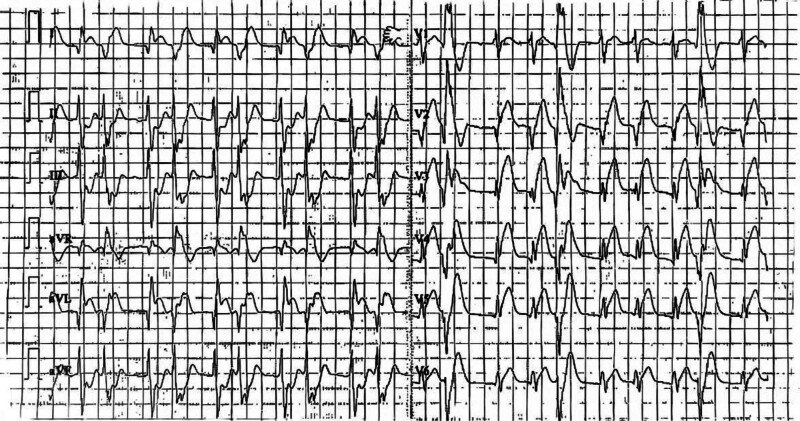
Twelve-lead electrocardiogram.

**Figure 2.**
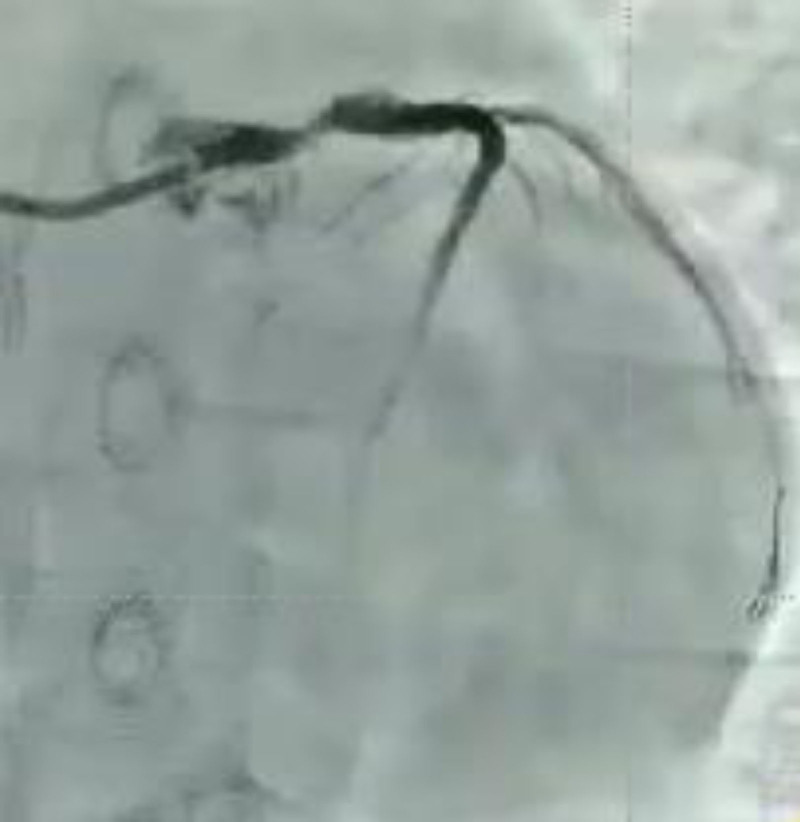
Coronary angiography.

**Figure 3.**
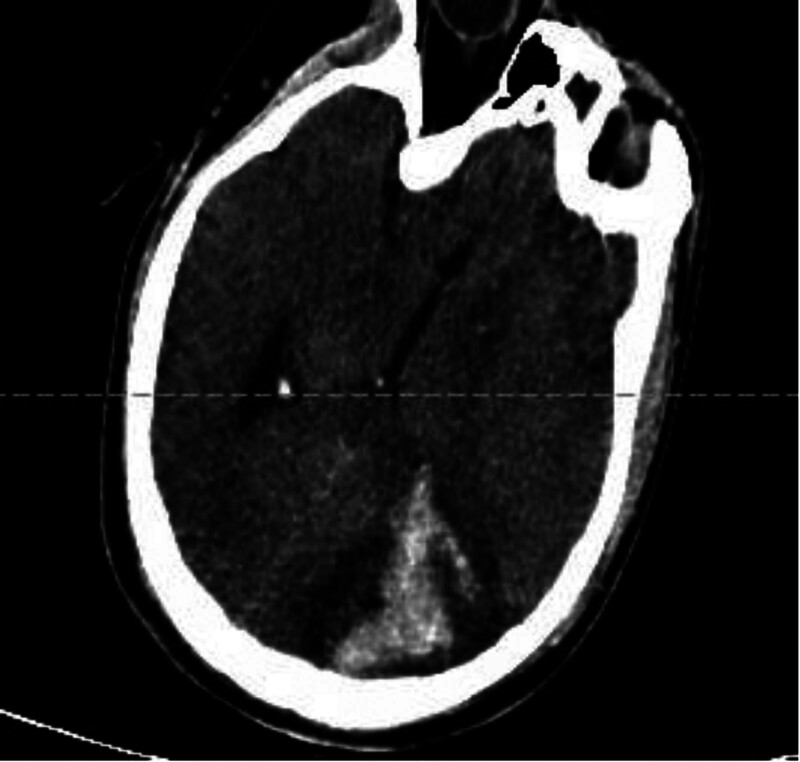
Cranial computed tomography.

The patient was a 16-year-old male student who presented to a local hospital with a chief complaint of persistent precordial chest pain lasting for more than 4 hours. He had no prior history of hypertension, diabetes mellitus, or congenital heart disease. There was no known family history of cardiovascular disease or hereditary disorders. The patient reported occasional alcohol consumption but denied any history of smoking or illicit drug use. He also denied experiencing significant psychological stress, irregular dietary habits, or chronic sleep deprivation. (The patient’s detailed diagnostic and treatment process, along with laboratory test results, are presented in Tables 1 and 2, Figs. 1–3.)

**Table 1:** Patient’s diagnosis and treatment timeline.

Time	Clinical course
Day 1 (onset day), ~01:00	The patient developed precordial chest pain after alcohol consumption, described as a dull, pressing pain that progressively worsened without relief. The pain was accompanied by nausea, vomiting, and profuse sweating
Day1, 02:00–04:00	He initially presented to a local hospital, where ventricular fibrillation occurred during ECG examination. Defibrillation was promptly performed, successfully restoring sinus rhythm. Vasopressors were administered to maintain blood pressure, though the patient remained in a confused state
Day 1, 05:20	The patient was subsequently transferred via emergency ambulance (120) to Puyang Oilfield General Hospital. On arrival, ECG revealed extensive anterior wall and high lateral ST segment elevation myocardial infarction, rapid atrial fibrillation, and frequent premature ventricular contractions (Fig. 1)
Day 1, 05:40	He was administered oral aspirin 300 mg and ticagrelor 180 mg. Emergency coronary angiography and percutaneous coronary intervention were performed. Angiography (Fig. 2) demonstrated complete occlusion of the left main coronary artery, with collateral retrograde flow to the left anterior descending and left circumflex arteries from the distal right coronary artery. Balloon dilation and thrombus aspiration were repeatedly attempted at the site of occlusion
Day 1, 07:10	Following the procedure, the patient was admitted to the intensive care unit. His blood pressure was 90/43 mm Hg while receiving norepinephrine at 0.05–0.1 µg/kg/min. Oxygen therapy via nasal cannula at 5 L/min resulted in a peripheral oxygen saturation (SpO₂) of only 81%. Echocardiography revealed severely reduced left ventricular function with an ejection fraction of 29% and diffusely decreased wall motion. Blood pressure remained difficult to stabilize despite high-dose vasopressors, and oxygenation failed to improve even with mechanical ventilation using 100% oxygen
Day 1, 09:55 to day 5, 19:27	The patient was subsequently placed on VA-ECMO, renal replacement therapy (dialysis), and received other supportive treatments
Day 7	Cranial computed tomography (CT) demonstrated patchy hemorrhage in the left occipital lobe (Fig. 3). To manage cerebral edema, mannitol, albumin, and furosemide were administered; anticoagulant and antiplatelet agents were discontinued and neutralized with protamine. A chest CT showed multiple patchy opacities and partial consolidation, with a clinical pulmonary infection score of 8. Sputum cultures isolated hypervirulent Klebsiella pneumoniae, for which tigecycline and polymyxin were initiated
Day 10	Subsequent cranial CT scans showed no significant change in the extent of hemorrhage
Day 16, 03:25	The patient’s condition continued to deteriorate, with blood pressure dropping to 45/20 mm Hg despite maximal vasopressor support. He progressed to a deep coma with bilaterally dilated pupils (6 mm), absent brainstem reflexes, and loss of spontaneous respiration. Despite ongoing intensive care, multiorgan failure and th rombocytopenia worsened. After discussion with the family, life-sustaining treatment was withdrawn, and the patient ultimately died

ECG = electrocardiogram, CT = computed tomography, VA-ECMO = venoarterial extracorporeal membrane oxygenation.

**Table 2:** Admission laboratory findings.

Parameter	Result	Unit
White blood cell	22.97	×10^9^/L
Neutrophil	20.13	×10^9^/L
Lymphocyte	2.42	×10^9^/L
Hemoglobin	164	g/L
C-reactive protein	89	mg/L
Procalcitonin	11.32	ng/mL
Platelet	324	×10^9^/L
Prothrombin time	25.5	S
Activated partial thromboplastin time	Undetectable	
Thrombin time	17.4	S
Fibrinogen	2.26	g/L
International normalized ratio	2.19	
Antithrombin III activity	80.0	%
Triglyceride	1.31	mmol/L
Total cholesterol	5.37	mmol/L
High-density lipoprotein cholesterol	0.93	mmol/L
Low-density lipoprotein cholesterol	3.48	mmol/L
Apolipoprotein A1	0.92	g/L
Apolipoprotein B	1.25	g/L
Myoglobin	>1200	ng/mL
High-sensitivity troponin I	>50	ug/mL
B-type natriuretic peptide	1622.20	ng/mL
Potassium	4.52	mmol/L
Sodium	138.0	mmol/L
Chloride	106.0	mmol/L
Alanine aminotransferase	161.0	IU/L
Aspartate aminotransferase	933.0	IU/L
Urea	4.42	mmol/L
Creatinine	87.5	μmol/L
Amylase	43	IU/L
Glucose	9.08	mmol/L
Hemoglobin A1c	5.40	%
Human immunodeficiency virus antibody	0.17	S/CO
Hepatitis C virus antibody	0.06	S/CO
Treponema pallidum chemiluminescence immunoassay	0.11	S/CO
Hepatitis B surface antigen	0.01	IU/mL

## 3. Discussion

Myocardial infarction (MI) is rare in patients younger than 20 years of age. Among young patients with acute coronary syndrome and obstructive coronary artery disease, smoking and dyslipidemia are the most common risk factors.^[4]^ Additionally, spontaneous coronary artery dissection, myocardial bridging, and coronary artery aneurysm are important etiologies of MI in adolescents. Notably, coronary artery aneurysm is a recognized cause of MI in this age group and may be associated with various underlying conditions, including Kawasaki disease, polyarteritis nodosa, connective tissue disorders such as Marfan syndrome, human immunodeficiency virus infection, and fungal or syphilitic infections.When evaluating MI in adolescents, toxic causes – particularly vasospasm induced by cocaine use – should also be carefully considered.^[5]^ A definitive diagnosis requires a comprehensive assessment of risk factors, clinical presentation, electrocardiographic changes, laboratory findings, and imaging studies.

Intracranial hemorrhage (ICH) is one of the most severe complications in adult patients receiving VA-ECMO. The reported incidence of ICH in this population ranges from approximately 12% to 21%, with a 1-month mortality rate reaching up to 86%. However, the underlying risk factors remain incompletely understood. Thrombocytopenia and low fibrinogen levels have been identified as independent risk factors.^[6]^ Studies suggest that the timing of resuming anticoagulation after cerebral hemorrhage critically affects outcomes: restarting too early increases the risk of recurrent bleeding, while delaying reinitiation elevates the risk of thrombotic complications. Current evidence indicates that resuming anticoagulation around 10 weeks after the onset of ICH may be optimal. In cases of minor hemorrhage without ongoing bleeding, or in instances where bleeding lesions are incidentally discovered on imaging with minimal prognostic significance, clinical strategies may include: temporarily discontinuing dual antiplatelet therapy for 5 to 7 days; stopping aspirin while continuing clopidogrel; or cautiously maintaining dual antiplatelet therapy under close monitoring.^[7]^ To date, the Extracorporeal Life Support Organization international multicenter registry has provided only limited evidence-based guidance regarding the type and size of ICH or the optimal management of coagulation in these patients. Ultimately, VA-ECMO therapy requires careful balancing between the risks of thrombosis and hemorrhage.

This case highlights the rare but severe occurrence of AMI in adolescents, emphasizing the importance of early recognition and prompt treatment, even in patients without traditional risk factors. However, this report has certain limitations, as a single case report, the findings are not broadly generalizable, and the fatal outcome may introduce reporting bias, potentially overstating the severity of AMI in young individuals. Nevertheless, it serves as a reminder that AMI should be considered in the differential diagnosis of chest pain in adolescents. Clinicians should maintain a high index of suspicion and act swiftly when evaluating such cases. Further studies are warranted to better understand the risk factors and optimal management strategies for early-onset AMI. (This case report has been approved by the Ethics Committee of Puyang Oilfield General Hospital. Ethics Approval Number: 2025-05-0021-E01.)

## Author contributions

**Supervision:** Ping Zhang.

**Writing** – **original draft:** Yuke Xie.

**Writing** – **review & editing:** Ning Yang.
